# Traditional medicine, environmental exposures, and cultural practices in cancer risk: insights from low- and middle-income countries

**DOI:** 10.3389/fonc.2025.1623895

**Published:** 2025-10-01

**Authors:** Thabiso Victor Miya, Rahaba Marima, Tebogo Marutha, Thifhelimbilu Emmanuel Luvhengo, Zilungile Mkhize-Kwitshana, Nkhensani Chauke-Malinga, Gugulethu Mazibuko, Zodwa Dlamini

**Affiliations:** ^1^ SAMRC Precision Oncology Research Unit (PORU), DSTI/NRF SARChI Chair in Precision Oncology and Cancer Prevention (POCP), Pan African Cancer Research Institute (PACRI), University of Pretoria, Hatfield, South Africa; ^2^ Department of Surgery, University of the Witwatersrand, Johannesburg, South Africa; ^3^ Department of Life and Consumer Sciences, College of Agriculture and Environmental Sciences, University of South Africa, Roodepoort, South Africa; ^4^ Papillon Plastic Surgery, Johannesburg, South Africa; ^5^ Faculty of Humanities, Department of African Languages, University of Johannesburg, Johannesburg, South Africa; ^6^ Wolfson Wohl Cancer Research Centre, School of Cancer Sciences, University of Glasgow, Glasgow, United Kingdom

**Keywords:** traditional medicine, environmental exposures, cultural practices, low- and middle-income countries, cancer risk, prevention strategies, dietary habits, mobile health

## Abstract

Cancer is a growing public health concern in low- and middle-income countries (LMICs), influenced by cultural practices, environmental exposures, and dependence on traditional medicine in addition to biological risk factors. Evidence from peer-reviewed publications published between 2010 and 2025 was combined in this narrative review. According to studies, traditional and complementary medicine (T&CM) is used by 35% to 79% of cancer patients in LMICs, which frequently delays biomedical treatment and complicates care. Over 2.4 billion people use biomass fuels for household air pollution (HAP), which has been associated with a two- to three-fold increased risk of lung cancer, especially in women. Furthermore, tobacco smoking contributes to about 2.7 million new cases of cancer in less developed areas each year, highlighting ongoing exposure to avoidable dangers. Findings show that the cancer burden in LMICs is further exacerbated by poor food storage, alcohol use, pesticide exposure, unregulated consumer chemicals, and stigma. These cultural and environmental factors must be addressed in preventative initiatives in addition to biological therapy. Strengthening T&CM regulations, enhancing food safety, upholding alcohol and tobacco legislation, lowering exposures at work and in the home, and introducing culturally-based education to dispel stigma and myths are among the top priorities. This is a narrative review rather than a systematic one; the goal is to map thematic evidence throughout Africa, Asia, and Latin America, providing insights for policy design. Integrated, context-specific, and community-driven approaches are required to eliminate inequities and promote equitable cancer control in LMICs.

## 1 Introduction

### 1.1 Background on cancer in LMICs

Cancer has become a serious global health concern because of increased mortality. Using the latest GLOBOCAN 2022 estimates (1), the global cancer burden in 2022 was ~19.90 million new cases and ~9.70 million deaths. Aggregating upper-, lower- and low-income countries (LMICs) shows they accounted for ~12.12 million incident cases (≈61% of the world total) and ~6.87 million deaths (≈71%), confirming that about 70% of cancer deaths and around 60% of new cases occur in LMICs (1). Early detection, cancer prevention, management, and treatment in high-income countries (HICs) have improved over the years. This has resulted in lower mortality and higher survival rates (2). However, this is different in low- and middle-income countries (LMICs). The World Bank’s classification of LMICs is based mostly on their gross national income (GNI) per capita. These categories are frequently used in studies on global health, development, and economics and are updated annually. According to the most recent World Bank classification, which was based on GNI per capita statistics from 2023, the GNI per capita in low-income countries is $1,135 or less per person, $1,136 to $4,465 for lower-middle-income countries. Lastly, $4,466 to $13,845 for upper-middle-income nations. Thus, LMICs encompass a broad range of economic and developmental circumstances but do not include high-income countries (3). Cancer cases in LMICs have risen sharply due to population growth and greater exposure to risk factors (4). Currently, 70% of cancer fatalities worldwide occur in LMICs, where outcomes are often poor. Furthermore, approximately 60% of new cancer diagnoses occur in LMICs (5, 6). The incidence of cancer in Africa has increased drastically in recent decades. This is due to a combination of variables, such as socioeconomic inequities, epidemiological shifts, and demographic changes (7). It is estimated that 2,000 people die of cancer daily in Africa. Furthermore, there are one million new instances of the disease each year. By 2030, this number is predicted to double (8). Because LMICs lack cancer infrastructure, early detection, and affordable treatment, survival rates remain low (9, 10). Treatment options for cancer depend on early diagnosis. However, because LMICs lack access to early detection, the mortality rates from cancer are higher than those from infectious diseases or malnutrition (10, 11). For instance, there are very few affordable and efficient breast cancer screening programs in LMICs (10).

Beyond the limitations of healthcare infrastructure, cultural practices play a significant role in shaping cancer prevention, risk, and treatment outcomes in LMICs. Traditional dietary habits, use of herbal remedies, tobacco and alcohol consumption, and reproductive behaviors contribute to cancer incidence. Cultural beliefs, stigma, and distrust of modern medicine hinder early detection and treatment efforts. Studies indicate that cultural barriers contribute to late-stage diagnoses and poorer survival rates in LMICs than in HICs (12).

**Figure 1:**
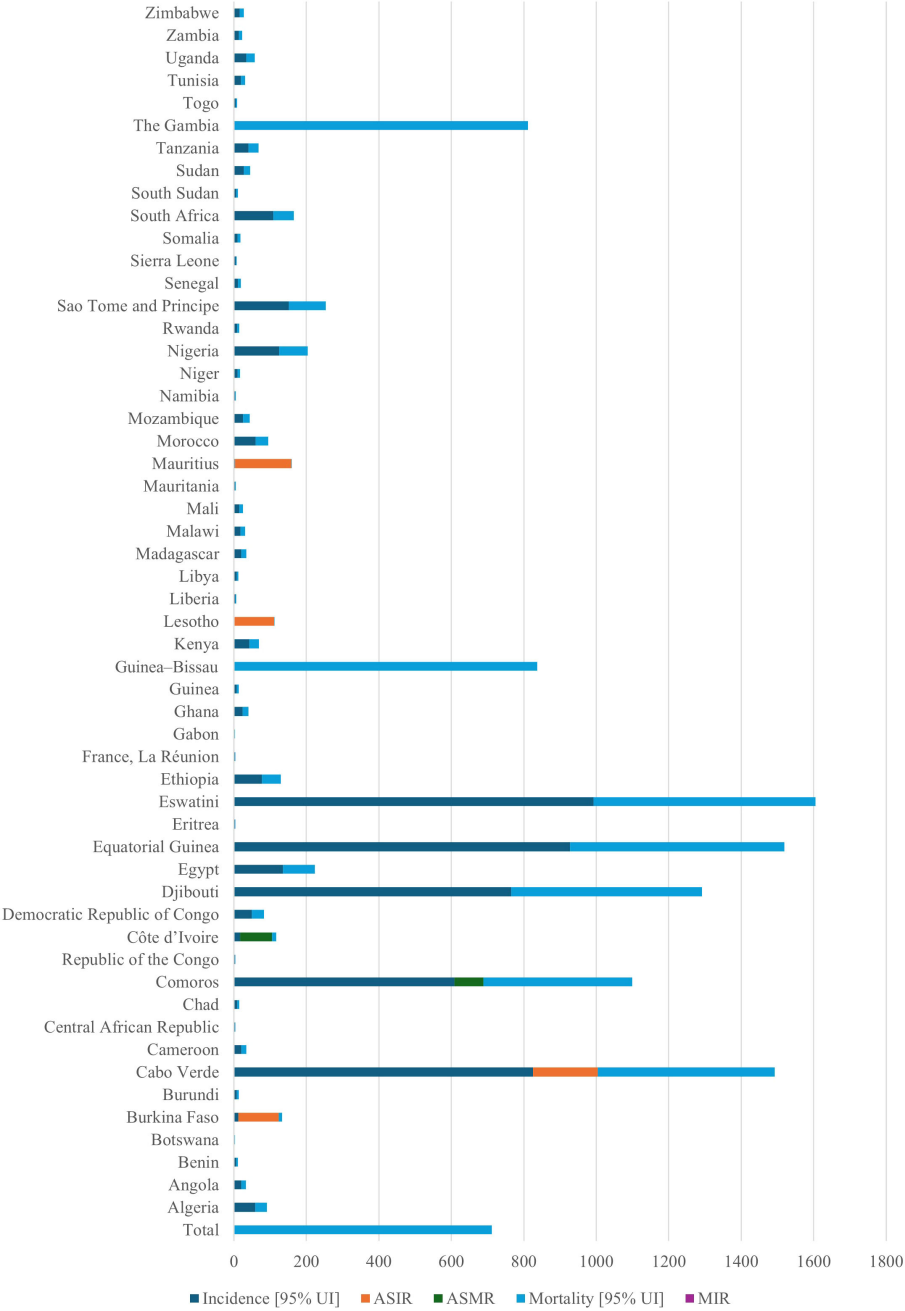
Total cancer incidence across 54 African nations in 2020. The figure demonstrates how cancer cases are unevenly distributed over the continent, with the highest burdens seen in countries with large populations like South Africa, Nigeria, and Egypt. The necessity for customized, nation-specific prevention efforts is highlighted by these discrepancies, which reflect demographic shifts, differences in risk factor exposure, and inequalities in the capacity of the health system. ASIR, age-standardized incidence rate; AMSR, age-standardized mortality rate. This is an original figure.

**Figure 2:**
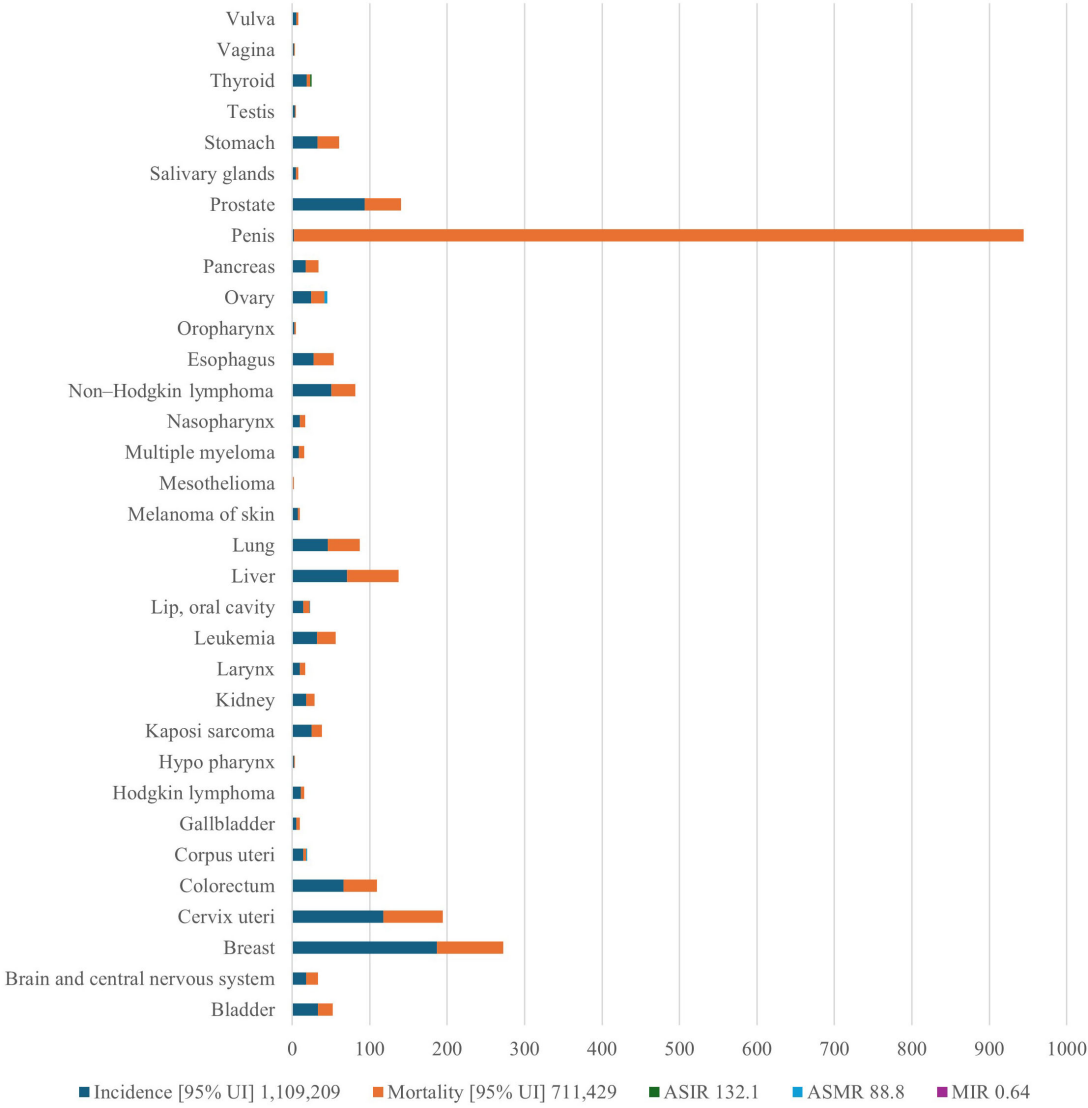
Prostate, liver, cervical, and breast cancers appear as the main causes of overall incidence, reflecting both regional risks (liver cancer linked to aflatoxin exposure, cervical cancer due to HPV prevalence) and global trends (increasing breast and prostate cancer linked to urbanization and lifestyle change, for example). The image highlights the necessity of prevention strategies that combine environmental risk reduction, lifestyle change, and infection management. HPV, human papilloma virus; ASIR, age-standardized incidence rate; AMSR, age-standardized mortality rate. This is an original figure.

Few studies have described the geographic distribution and drivers of cancer in LMICs. This is despite the rising cancer cases and death rates in these nations. Sharma et al. (13) used spatial epidemiology to analyze the cancer burden in Africa, linking socioeconomic status to cancer risk and mortality (13). Research indicates that in 2020, there were 711,429 cancer-related deaths and more than 1.1 million new cases. **Figure 1** demonstrates the considerable variation in cancer incidence across the African continent, with South Africa, Nigeria, and Egypt contributing a disproportionately high proportion of cases. These patterns largely reflect underlying population distributions. These discrepancies are a result of shifting demographics, changes in risk factor exposure, and inequalities in the capacity of the healthcare system. The findings highlight the necessity of investigating how access to care, environmental exposures, and cultural practices interact to influence cancer burden in various ways across national contexts. The distribution of the cancer burden by type in Africa is shown in **Figure 2**, where the most important causes of total incidence are liver, prostate, cervical, and breast cancers. This distribution reflects both regional factors (e.g., liver cancer associated with aflatoxin exposure and cervical cancer owing to HPV prevalence) and global trends (e.g., the increasing incidence of breast and prostate cancer linked to urbanization and lifestyle changes). These findings highlight the significance of adjusting preventive measures to Africa’s distinct cancer profile, where environmental and infection-related malignancies continue to be more common than in high-income areas.

The mortality-to-incidence ratio (MIR) showed a strong inverse correlation with the human development index (HDI). Additionally, it was estimated that by 2040, the cancer burden in Africa will rise to 2.1 million new cases and 1.4 million fatalities. This is due to shifting demographics and population growth alone, assuming that age-specific cancer rates remain the same. The study estimated that the cancer burden will rise further by 2040. This is despite the study not having comprehensive cancer control strategies that address both biomedical and sociocultural factors implemented across Africa’s LMICs (13). According to a different study that examined the prevalence of childhood malignancies in 183 nations, LMICs bear almost 80% of the global burden of childhood cancer (12).

The incidence and death rates of cancer are rising across Asia. Mubarik et al. (14) determined the risk-attributable deaths from breast cancer and forecasted the future death risk from breast cancer in East and South Asian (ESA) nations (14). The ESA region is expected to see an increase in breast cancer-related deaths over the next ten years. Between 1990 and 2030, the age-standardized mortality rate from breast cancer is projected to rise by 35.0% in South Asia and 7.0% in East Asia. Additionally, low-to-middle socio-demographic index countries in the region have a higher risk of breast cancer death. To address the growing incidence of breast cancer in ESA nations, early detection, prompt and affordable treatment, and increased awareness are recommended (14). Another study on the prevalence of breast cancer in Asia revealed that the death rates were higher. Additionally, survival rates are poorer in LMICs across Asia because of late-stage diagnosis, a lack of screening choices, and treatment options (11).

### 1.2 Cultural practices and cancer

**Figure 3:**
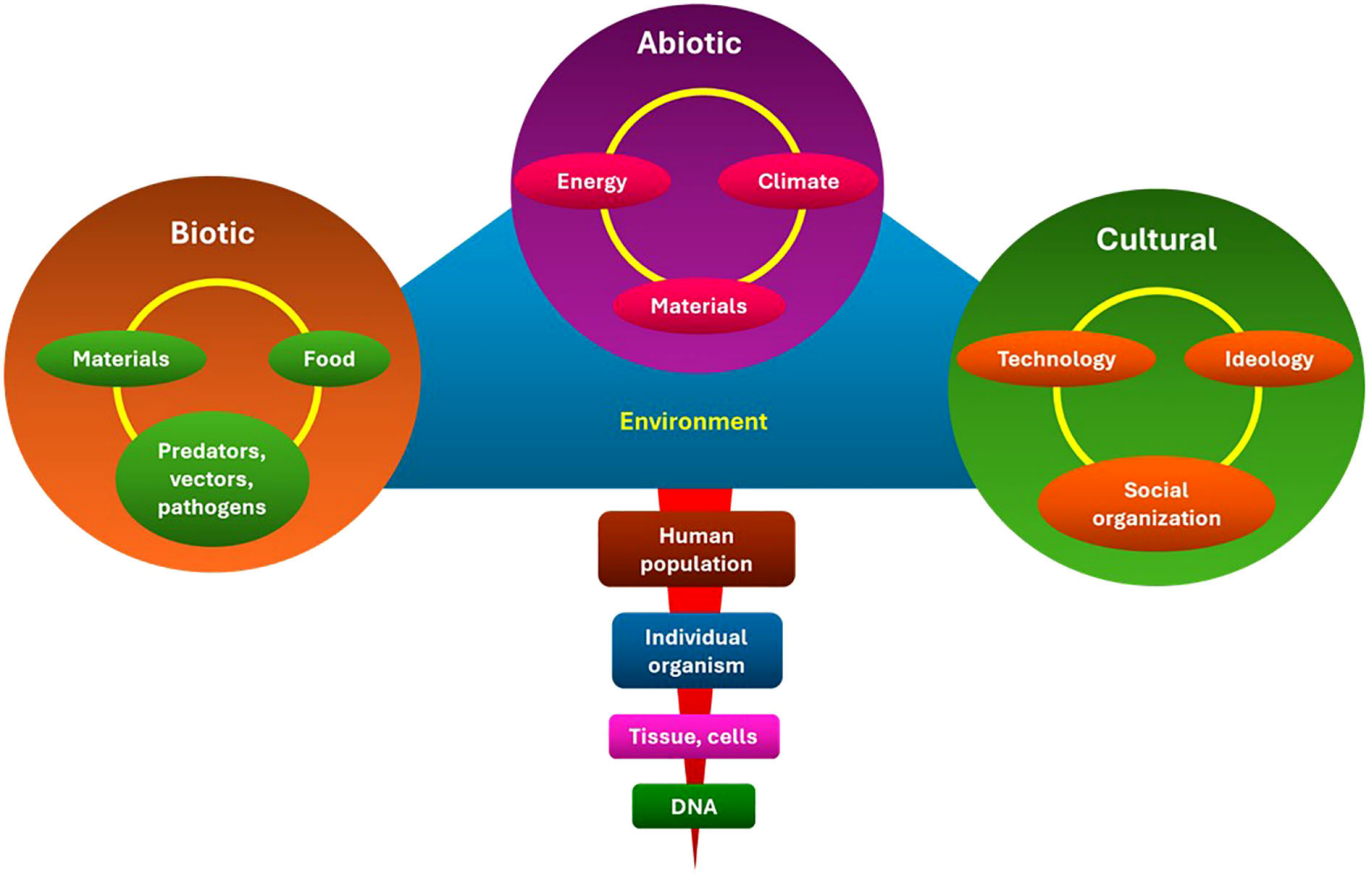
Ecological model of cancer risk in LMICs. The model illustrates how cancer outcomes are influenced by the interaction of biotic (genetic, infectious), abiotic (environmental, occupational, chemical), and cultural (diet, T&CM, stigma) factors. It highlights the necessity of multifaceted, integrated approaches to control and prevention. LMICs, Low- and middle-income countries; T&CM, Traditional and complementary medicine. This is an original figure.

Culture is the organizational structure of life that ensures the survival and well-being of people. It helps individuals find purpose and meaning in their lives and communicate care. It was created to ensure the well-being and survival of its members. This system consists of lifestyles, values, and beliefs that allow for effective adaptation within abiotic and biotic geographic niches by utilizing accessible financial and technological resources (15). This ecological model is depicted in **Figure 3**, which demonstrates the interplay between biotic (genetic predisposition and infection), abiotic (environmental exposures and occupational hazards), and cultural (diet, T&CM use, and stigma) factors that affect cancer incidence and survival. This model emphasizes the need for cancer control measures to target overlapping and reinforcing determinants of health rather than relying solely on single-risk approaches.

For instance, every cultural group uses various tools to control its surroundings for shelter and food. This makes emotional and cognitive sense of the chaos of its surroundings and find institutional and interpersonal social interactions that are structured and meaningful to promote the well-being of its members. Crucially, culture offers a means of making sense of life events through its worldview or construction of reality, particularly in difficult times, such as when a person is diagnosed with cancer (16). This thinking makes illness and death easier to understand and deal with through specific values, beliefs, and customs. It influences how a person’s social network expresses concern, offers social support, and ensures safety. Additionally, it influences how one should behave emotionally and behaviorally toward an illness. Therefore, the majority of culturally prescribed and proscribed factors that affect gene expression, disease prevalence, and health status are related to diet, marital laws, social roles, and means of subsistence (17). The seven nested elements of culture that define its composition and purpose are listed in **Table 1** (18).

**Table 1:** Seven nested layers of culture in no particular order.

The seven nested layers of culture	
1	Economy
2	Language
3	Technology
4	Environment
5	Social Structure
6	Beliefs and Values
7	Religion/World View

These include the environment, economy, and technology.

Therefore, culture is not just a set of values and beliefs that are interchangeable with the dominant Northern European-American culture; rather, it is a multidimensional, multilevel, dynamic, biopsychosocial, and ecological system in which a population lives (19). Cultures evolve differently because of these variables, which change and adapt dynamically in response to social, political, and geographical conditions. Any attempt to incorporate the idea of culture into medical practice needs to be evaluated both throughout time and at each level of the model (16). The distinction between the terms race, racism, ethnicity, and culture allow us to discuss their contributions to health inequalities. This also acknowledges that their interaction with other elements that make up the social determinants of well-being ultimately affects health. At best, the existing imprecision in the use of these terms leads to false assumptions about specific people (20, 21), making it difficult to understand how each notion affects cancer outcomes throughout the entire spectrum of care (22).

One of the fundamental pieces of information required for any medical visit is the social history section of the patient’s history and physical examination. By incorporating important information at all seven levels of culture (**Table 1**), Oncologists can better understand how culture affects the meaning and experience of cancer and its treatment for patients and their families. This is despite the fact that some aspects of culture have already been evoked in social history. Oncologists may need to spend more time during initial consultations with patients to obtain a more thorough sociocultural history. However, this extra time and effort could end up saving time by fostering a relationship of trust and allowing for a more candid discussion of the patient’s viewpoint, preferences, and cultural beliefs. The initial commitment would probably avoid many cultural miscommunications and disputes, resulting in more culturally aware and considerate cancer treatment that would eventually be more economical (16). Therefore, this review examines the relationship between cultural practices and cancer risk in LMICs, emphasizing how traditional factors such as tobacco use, alcohol use, traditional medicine use, dietary patterns, cancer myths, and stigmas increase the risk of cancer. This review also discusses cancer prevention strategies, such as policy implementation, early screening, cancer awareness, education, and funding.

Through a variety of ecological rings, including biotic, abiotic, and cultural variables, cultural practices interact with cancer risk. Aflatoxin-contaminated traditional foods in SSA are one example of how dietary practices inside the biotic ring shape microbial exposures that may influence cancer pathways (23, 24). The physical environment is captured by the abiotic ring, where activities such as burning biomass fuel indoors expose people to home air pollution, a known lung carcinogen that disproportionately affects women in rural areas (25). The cultural ring depicts societal customs and norms that influence health behaviors, such as chewing betel quid, which significantly increases the risk of oral cancer in South and Southeast Asia (26, 27).

The evidence in Sections 2 and 3 was chosen and arranged according to this ecological framework. The studies were categorized based on the ring that was most pertinent to the exposure pathway: cultural (e.g., social practices, gendered behaviors), abiotic (e.g., environmental and occupational carcinogens), and biotic (e.g., infectious agents, food exposures). This framework preserved analytic coherence across a range of exposures while guaranteeing that the review included both proximal and distal cultural factors of cancer risk.

### 1.3 Search strategy

This review employed a narrative synthesis approach to collect evidence on cultural behaviors, environmental exposures, and traditional medicine in connection to cancer risk in LMICs. We did electronic searches in PubMed, Web of Science, and Google Scholar between March 15 and March 30, 2025. The searches were restricted to English-language publications.

Boolean search phrases included:

(“traditional medicine” OR “complementary medicine” OR “herbal remedies”) AND (“cancer” OR “oncology”) AND (“low- and middle-income countries” OR LMIC OR Africa OR Asia OR Latin America)
(“environmental exposures” OR “household air pollution” OR “occupational hazards”) AND (“cancer risk” OR “carcinogen*”) AND (LMIC OR “developing countr*”)

Filters: Publication years 2010–2025; Article types: original research, systematic reviews, narrative reviews, and peer-reviewed policy reports.

#### 1.3.1 Screening process

There were two stages to the screening procedure. Initially, two reviewers independently checked abstracts and titles for appropriateness. Second, the whole texts of the relevant articles were obtained and examined. Disagreements were handled by discussion, and if consensus was not established, a third reviewer adjudicated.

#### 1.3.2 Handling of grey literature

Editorials, comments, and conference abstracts that were not subjected to peer review were not included. Nonetheless, because of their significance for exposure classification and cancer prevention policy, policy, and technical reports from significant international organizations (such as the WHO, UN, and IARC) were included where immediately pertinent.

#### 1.3.3 Study selection outcome

**Figure 4:**
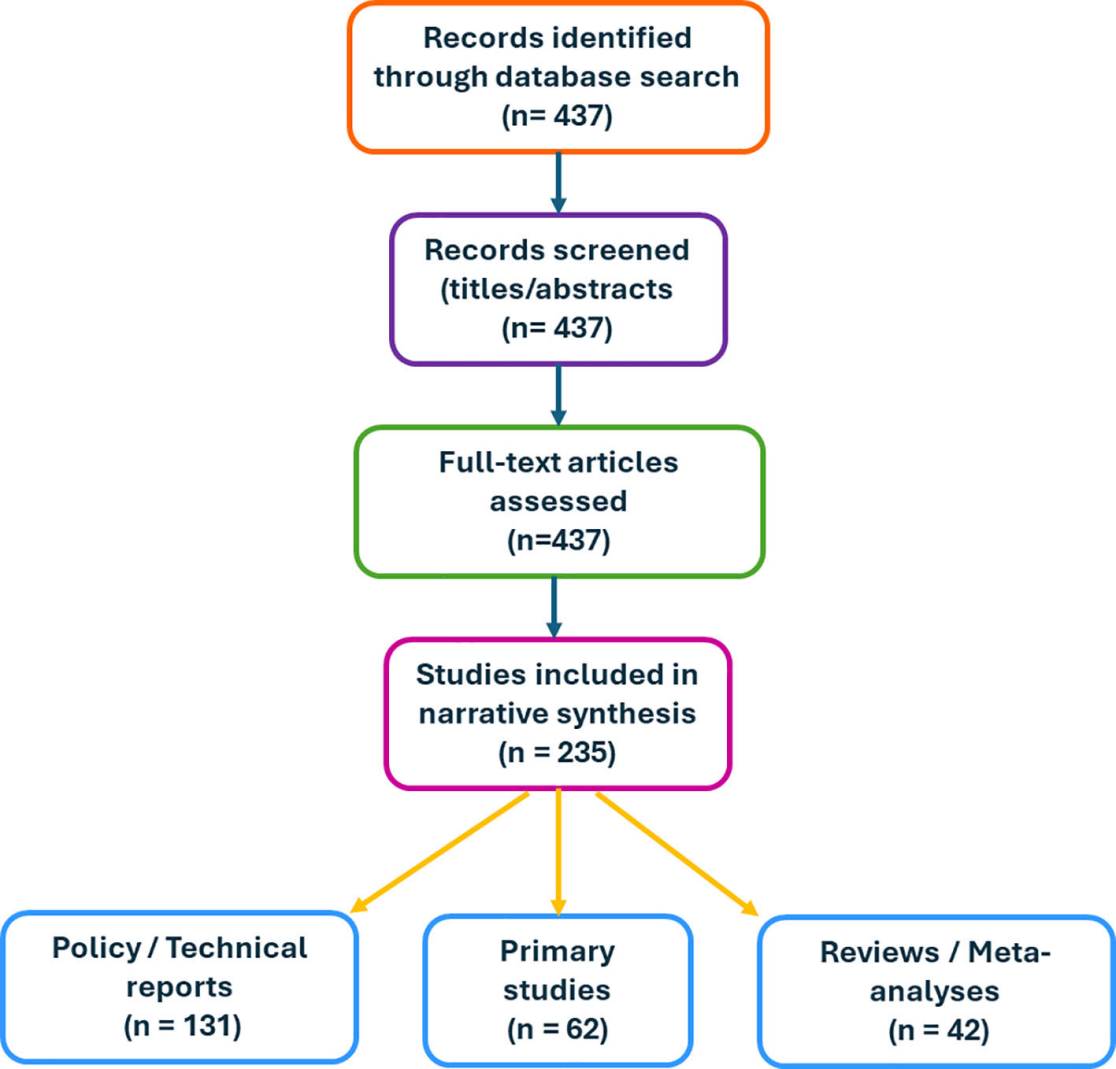
Flow diagram of the search and selection process. A total of 437 records were identified through database searching (2010–2025). After title and abstract screening, full-text articles were assessed for eligibility. In total, 235 studies were included in the narrative synthesis, comprising 131 policy/technical reports, 42 reviews or meta-analyses, and 62 primary studies. This is an original figure.

Following deduplication, 437 records were found. Furthermore, 235 peer-reviewed publications were included after screening, comprising 131 policy/technical reports, 42 reviews or meta-analyses, and 62 main investigations (**Figure 4**).

#### 1.3.4 Limitations of the search

We did not follow PRISMA reporting guidelines or conduct a formal risk-of-bias assessment because this was a narrative evaluation rather than a systematic one. It is possible that pertinent material published in local languages was overlooked by limiting inclusion to English-language studies. Furthermore, inconsistent database coverage in LMIC settings could skew results in favor of research from international partnerships or higher-impact journals.

## 2 Examples of cultural practices contributing to cancer risk

### 2.1 Traditional dietary practices

Dietary habits in LMICs are influenced by cultural norms, food availability, and socioeconomic circumstances. Although many traditional diets are built on nutrient-dense whole foods, poor food preservation and storage techniques greatly increase the risk of cancer.

#### 2.1.1 Prevalence and exposure data

The WHO-recommended maximum levels of aflatoxin (20 µg/kg) are exceeded in 25–65% of maize samples examined in sub-Saharan Africa, with contamination being highest in humid and poorly ventilated storage conditions (28, 29). Fumonisin contamination is also common; according to surveys conducted in East and Southern Africa, 40–60% of household maize samples have levels above the provisional maximum tolerated daily intake set by the Joint FAO/WHO Expert Committee on Food Additives (30).

#### 2.1.2 Cancer risk estimates

Exposure to aflatoxin B1 is closely associated with hepatocellular carcinoma (HCC). According to a meta-analysis, those who have been exposed to high levels of aflatoxin have a 5.5 percent chance of getting HCC (95% CI: 2.9–10.5), and their risk increases when they also have a chronic hepatitis B infection (24). According to pooled data from high-exposure regions in China and South Africa, fumonisin B1 exposure has also been linked to an elevated risk of esophageal cancer, with an odds ratio (OR) of 2.3; 95% CI: 1.4–3.7 (31). These results highlight the fact that exposure to dietary carcinogens in LMICs is ubiquitous and clinically meaningful rather than accidental.

#### 2.1.3 Feasibility and policy considerations

For food-environment interventions to be successful in LMICs, they must be culturally appropriate. According to data, using triple-layer hermetic storage bags instead of standard woven sacks can minimize aflatoxin contamination in maize by as much as 60% over six months (32). In a similar vein, research in rural East Africa has shown that solar-powered cold-chain storage for perishable commodities can reduce spoiling and fungal contamination by 30 to 50% (33). However, market incentives for aflatoxin-safe crops, cost, and conformity to conventional storage standards are necessary for adoption.

LMICs face the combined burden of undernutrition and mycotoxin-related cancers, in contrast to HICs, where obesity predominates diet-related cancer risk. To ensure that policies align with local food practices and encourage safe agricultural trade, effective interventions necessitate both technological solutions (such as enhanced storage, fortification, and cold-chain access) and cultural adaptation.

### 2.2 Use of herbal remedies and traditional medicine

Traditional and complementary medicine (T&CM) use is deeply entwined with the healthcare and cultural contexts of LMICs. T&CM, which has its roots in millennia of indigenous wisdom, is frequently used as a primary and supplemental method of treating cancer and other illnesses. It includes a variety of fields, including nutritional therapies, herbal remedies, and spiritual healing (34). T&CM is widely employed for cancer prevention, diagnosis, and treatment in many LMIC situations, which brings with it both potential and major obstacles.

In situations with limited resources, T&CM is often the initial course of treatment for patients. According to surveys, between 34.5% and 79% of cancer patients in sub-Saharan Africa (SSA) use T&CM, either alone or in conjunction with traditional treatments (35–37). According to reports, 35% to 61% of cancer patients in Iran and Turkey seek alternative medicines, demonstrating a similarly high utilization rate (38, 39). The main causes of this dependence are cultural views, faith in traditional healers, lack of access to biomedical facilities, and financial limitations (40, 41). Although T&CM fosters community trust and offers culturally competent treatment, its uncontrolled usage is dangerous. Herbal treatments frequently lack thorough testing for toxicity, effectiveness, and pharmacokinetic interactions with traditional cancer treatments, despite claims of safety (42). Hepatotoxicity, nephrotoxicity, anemia, and severe herb–drug interactions are among the documented side effects that can make cancer patients more vulnerable (43, 44). Furthermore, patients may hide their use of T&CM from healthcare professionals out of concern for their approval, which makes risk reduction and treatment planning more difficult (42, 45). Up to 70% of patients in some areas first seek advice from traditional healers, frequently postponing biomedical treatment until the disease has progressed, which jeopardizes early identification and survival results (46, 47).

Key herbal preparations in oncology across chosen locations are summarized in **Table 2** to give a better understanding of the most popular herbal medicines, their possible toxicities, and known drug–herb interactions. T&CM is still a vital healthcare tool, nevertheless, emphasizing the necessity for integrative approaches as opposed to simple rejection. To improve safety, research, and quality control in T&CM, several LMICs, such as Ghana, South Africa, Ethiopia, and Tanzania, have started implementing regulatory frameworks (48, 49). However, progress is nevertheless constrained by persistent deficiencies in practitioner training, regulatory monitoring, and scientific evidence. The development of culturally sensitive cancer control strategies should strike a balance between respect for customs and patient safety and evidence-based treatment. Priorities include organized communication between traditional and biomedical practitioners, training programs for communities and healthcare professionals, and a methodical assessment of popular herbal medicines. T&CM will continue to function mainly unregulated in the absence of such regulations, which could jeopardize cancer prevention and treatment in LMICs (34, 50).

**Table 2:** Common herbal preparations in oncology, known toxicities, and drug–herb interactions.

Region/country	Herbal preparation	Common use in oncology	Known toxicities	Known Drug–Herb interactions (level of evidence)	References
East Africa (Ethiopia, Tanzania)	Ocimum spp. (Holy basil)	Symptom management, immunomodulation	Hypoglycemia, mild liver enzyme elevation	Potential additive effect with anticoagulants (low-moderate)	(34, 43, 44)
SSA (Uganda, Kenya)	Warburgia ugandensis	Anti-inflammatory, symptom relief	Nephrotoxicity (high doses)	No high-quality clinical interaction data (low)	(34, 43, 44)
SSA (Nigeria, Ghana)	Prunus africana	Prostate cancer support	Hepatotoxicity, gastrointestinal upset	CYP3A4 inhibition; may increase docetaxel toxicity (moderate)	(34, 43, 44)
SSA/Global	Sutherlandia frutescens	Symptom relief, cachexia	Mild nausea, hepatotoxicity in rare cases	Unknown interactions; limited clinical evidence (low)	(34, 43, 44)
Middle East (Iran)	Curcuma longa (turmeric)	Chemoprevention, adjuvant therapy	GI upset, hyperbilirubinemia	May reduce absorption of tamoxifen (low-moderate)	(34, 43, 44)
Asia (China, India)	Ganoderma lucidum (reishi)	Immune support, adjunct therapy	Mild hepatotoxicity	May potentiate anticoagulants (low)	(34, 43, 44)
Turkey	Glycyrrhiza glabra (licorice)	Fatigue, inflammation	Hypertension, hypokalemia	May increase corticosteroid effects (moderate)	(34, 43, 44)

Evidence levels are determined using a combination of clinical case reports, pharmacokinetic research, and systematic reviews. SSA, Sub-Saharan Africa.

#### 2.2.1 Policy considerations for T&CM in oncology

Safety and Quality (Pharmacovigilance): Regulatory frameworks should mandate standardized quality control for herbal products, including contamination testing, consistent dosing, and post-market monitoring for adverse effects. Pharmacovigilance systems must be integrated with national cancer registries in order to monitor toxicity and herb-drug interactions.
Training and Certification: T&CM practitioners should complete official training and certification programs that emphasize safe clinical practice, toxicology recognition, and collaborative referral protocols. Certification promotes responsibility and confidence among biomedical providers.
Referral and integration models: Models range from co-located clinics to formalized referral channels in which traditional healers identify high-risk patients and collaborate with oncologists. Formal integration within national health systems promotes communication between T&CM and biomedical practitioners, shortening diagnostic delays and limiting risky therapy overlap.

By distinguishing regulating processes in these domains, LMICs can maintain T&CM’s cultural relevance while ensuring patient safety and promoting evidence-based oncology care.

### 2.3 Tobacco use and cancer risk

#### 2.3.1 Tobacco use

Tobacco remains the leading preventable cause of cancer worldwide. According to GLOBOCAN 2020, tobacco use induced around 2.5 million new cancer cases (13%) and 1.8 million deaths (18%) globally in that year (51). These statistics are constant across genders and regions, while the majority of the global burden is concentrated in LMICs, which reflect both large populations and inadequate control measures (52, 53).

While global tobacco consumption has declined modestly in high-income countries (HICs) due to stringent public health policies, the burden of tobacco use is increasingly shifting toward LMICs, where over 80% of the world’s 1.3 billion tobacco users now reside (54, 55). Several variables are responsible for these changes. The tobacco industry uses aggressive marketing strategies to target LMICs, taking advantage of regulatory gaps, lax enforcement, and growing disposable incomes (56). Simultaneously, social customs and cultural norms support tobacco use, frequently normalizing smoking and smokeless tobacco use in local communities (57). For instance, despite their well-established links to pancreatic, esophageal, and oral malignancies, smokeless tobacco products such as betel quid, gutkha, and khaini are extensively used and profoundly ingrained in cultural practices in South Asia (58).

Economic inequality is also important. Low socioeconomic groups have a disproportionately high rate of tobacco use and are more likely to smoke and be at risk for tobacco-related health issues. Ironically, despite having the largest disease burden, these populations frequently have the least access to early cancer detection services and cessation support (55, 59). The World Health Organization (WHO) Framework Convention on Tobacco Control (FCTC), adopted in 2003, has not been consistently implemented in LMICs. Owing to political opposition, worries about the effects on the economy, and ongoing corporate meddling, proven policies such as taxes, advertising bans, graphic health warnings, and smoke-free environments are frequently not strictly enforced (54, 60). There is significant potential for price-based interventions in LMICs, as evidence suggests that a 10% rise in tobacco prices can reduce tobacco use by 4–8%, especially among youth and low-income groups (60). **Table 3** summarizes the distribution of tobacco-related malignancies. Importantly, the table includes all cancers in which tobacco is a risk factor (lung, bladder, head and neck, esophageal, cervical, pancreatic, stomach, kidney, etc.), not just the attributable case portion.

**Table 3:** Tobacco-related malignancies (all sites causally linked to tobacco) and their GLOBOCAN 2020 incidence and fatality counts.

	Incidence		Mortality	
	Men	Women	Men	Women
Less-developed countries counts	2,697,000	1,651,000	2,212,000	1,242,000
More developed countries counts	1,490,000	848,000	980,000	568,000
Global counts	4,186,000	2,499,000	3,193,000	1,811,000

The values represent overall site burden, not tobacco-attributable fractions.

According to the data in **Table 2**, less developed areas have higher mortality-to-incidence ratios and account for most tobacco-related cancer cases. This is because these regions have poorer health systems, fewer treatment options, and later-stage diagnoses (61). The growing prevalence of tobacco-related malignancies in LMICs poses a threat to further increase global health inequities unless policies are implemented decisively and culturally appropriate interventions are implemented. To save millions of preventable cancer deaths, tobacco use in LMICs must be addressed using comprehensive methods that combine public education, regulatory enforcement, and culturally appropriate quitting support.

##### 2.3.1.1 Best-buy policy measures and expected impact

WHO defines a set of “best buys” for tobacco reduction, including information-based, regulatory, and economic measures, all of which have a proven track record of success:

- Excise taxation: According to Chaloupka et al. (62), the price elasticity of demand for tobacco in LMICs normally falls between -0.4 and -0.8, which means that a 10% price rise lowers consumption by 4-6 percent. The most receptive demographics are young and low-income (62). The WHO recommends excise taxes to be at least 75% of retail prices, however only 15% of LMICs implement them despite compelling evidence (63).

- Pictorial health warnings: Large visual health warnings that take up at least half of the pack decrease initiation and enhance attempts to quit. According to meta-analyses, there is a considerable rise in both intention to quit and understanding of the hazards; the effects are more pronounced in populations with poorer baseline health literacy (64). However, a lot of LMICs have tiny, text-only labels or no visual warnings at all.

- Smoke-free laws: Complete prohibitions on smoking in public and workplaces lower secondhand smoke exposure by 80–90% in venues under observation and are linked to sharp drops in hospitalizations for respiratory disorders and acute coronary events (65). Strong mechanistic and exposure-reduction evidence supports long-term benefits, but longer follow-up is necessary for cancer outcomes. Partial exemptions are still allowed in many LMICs, which decreases their efficacy.

Millions of cancer mortalities from tobacco use could be avoided if these steps are fully implemented. The tobacco industry’s meddling, lax enforcement, and financial dependence on tobacco profits, however, prevent policy adoption in LMICs, where implementation gaps are still significant.

#### 2.3.2 Smokeless tobacco consumption

The use of smokeless tobacco (SLT) contributes significantly to the cancer burden in LMICs, although being frequently disregarded. In contrast to smoking, SLT products are applied directly to the cheek, nasal cavity, or oral cavity, exposing nearby tissues to high levels of carcinogens (57, 66). Particularly in South and Southeast Asia, where betel quid, gutkha, and khaini are consumed as part of hospitality and everyday routines, its use is ingrained in cultural and social practices (58). The IARC has categorized SLT as carcinogenic to humans, despite the general perception that it is less harmful than smoking. SLT has a strong epidemiological association with pancreatic, pharyngeal, esophageal, and oral cavity malignancies (59, 67). For instance, regular SLT users are at least twice as likely to get oral cancer than non-users, and the risk increases further in areas where chewing betel quid is common (67).

There are other health hazards associated with SLT use. Negative outcomes such as stillbirth, low birth weight, and neonatal mortality are linked to its use during pregnancy (58). However, in LMICs, SLT regulation is still uneven. While all tobacco products are included in the WHO Framework Convention on Tobacco Control (FCTC), enforcement of the FCTC is disproportionately concentrated on cigarettes (54). By aggressively selling SLT as a safer and more socially acceptable alternative, tobacco corporations take advantage of this gap, particularly with women and young people (55, 56). Furthermore, since SLT is widely accessible and reasonably priced, it is more likely to be used by lower socioeconomic groups, who already have limited access to cancer prevention and treatment (59). Using GLOBOCAN 2020 projections (68). **Table 4** shows the global incidence and mortality statistics for all malignancies associated with tobacco use (smoked and smokeless). Rather than the more limited sample of attributable cases, these numbers include all malignancies linked to tobacco smoking.

**Table 4:** The global incidence and mortality rates for tobacco-related malignancies (smoked and smokeless), according to GLOBOCAN 2020 (68).

	Incidence (women)	Incidence (men)	Mortality (women)	Mortality (men)
More developed	870,000	1,530,000	590,000	1,020,000
Less developed	1,810,000	2,970,000	1,390,000	2,450,000
Global total	2,680,000	4,500,000	1,980,000	3,470,000

These data cover all tobacco-related cancers, not just those induced by tobacco use.

The necessity to broaden tobacco control measures beyond cigarettes is shown by the continuance of SLT use. High efficacy is demonstrated by WHO FCTC best-buy interventions. A 10% rise in tobacco excise taxes, for example, lowers consumption by 4% to 8%, especially among young people and those with low incomes (60). Comprehensive smoke-free regulations reduce acute coronary hospitalizations by 10–20% during the first year, with long-term advantages for cancer prevention (69). Pictorial health warnings improve quit attempts by 20–30% (54). However, due to conflicting political and economic agendas, industry meddling, and lax enforcement, LMICs have significant implementation gaps. To lessen the rising prevalence of tobacco-related malignancies in LMICs, SLT and smoked tobacco must be addressed in culturally appropriate ways.

### 2.4 Alcohol consumption

Alcohol consumption is a leading cause of cancer worldwide and is becoming more widely acknowledged as a serious risk factor in LMICs. It is thought to be responsible for about 4% of all new cancer cases globally each year and is closely linked to malignancies of the female breast, pharynx, larynx, liver, esophagus, colorectum, and oral cavity (23, 70, 71).

#### 2.4.1 Cultural contexts and illicit alcohol

Drinking alcohol is ingrained in social and cultural customs in many LMICs. Commonly enjoyed outside of official regulatory frameworks are locally produced drinks, including palm wine, sorghum beer, and home-distilled spirits. WHO estimates that illicit or unrecorded alcohol accounts for 30 to 50 percent of total alcohol use in South Asia and SSA (72). Higher ethanol concentrations and impurities like methanol or acetaldehyde are commonly found in these goods, making it more difficult to estimate cancer risk and implement policies. Since a large portion of alcohol consumption takes place outside of official markets, the widespread usage of unrecorded alcohol also lessens the impact of traditional taxing schemes.

#### 2.4.2 Policy responses and measured outcomes

Despite these limitations, there have been successful alcohol policy interventions in LMICs. For example:

Between 2002 and 2012, South Africa introduced stricter alcohol advertising restrictions and increased excise taxes, resulting in a ~16% decrease in per capita consumption and reduced alcohol-related traffic injuries and liver disease mortality (73).
Brazil implemented a comprehensive alcohol control program that included restrictions on supply, advertising bans, and increased taxes. Between 2006 and 2012, surveys revealed a decrease in heavy episodic drinking among adults from 29% to 24%, with corresponding decreases in liver cirrhosis mortality (74).

#### 2.4.3 Implications for cancer prevention

These examples show that, even in situations with high levels of cultural embeddedness, well-executed, multifaceted methods can change consumption patterns. However, the effectiveness of traditional measures like raising the excise tax is limited by the vast informal alcohol production. Therefore, community education, gender-sensitive messaging, and systems to control or replace traditional brews must all be included in policy frameworks that target both the commercial and unrecorded alcohol markets.

In summary, the prevalence of unrecorded alcohol in LMICs creates a structural regulatory problem in addition to the biological carcinogenic risk associated with alcohol usage. The incidence of alcohol-related cancer in LMICs is expected to keep rising unless interventions are adapted to cultural norms and informal markets. Evidence from nations like Brazil and South Africa shows that focused, properly implemented interventions can lower hazardous consumption and produce quantifiable health benefits, but they must be tailored to local contexts where unrecorded alcohol use is still common.

### 2.5 Occupational and environmental exposures as contributors to cancer risk

#### 2.5.1 Occupational carcinogen exposure in traditional livelihoods

##### 2.5.1.1 Silica (stone-cutting, quarrying, small-scale mining)

Crystalline silica is known to induce lung cancer. Relative risks of lung cancer increased by up to 1.6 in the highest exposure group as compared to the lowest, according to a pooled analysis of ten occupational cohorts that demonstrated a strong exposure–response (75).

##### 2.5.1.2 Pesticides (smallholder farming, artisanal application)

Prostate and lympho-hematopoietic cancers are associated with specific pesticide classes. A 45% higher incidence of non-Hodgkin lymphoma was found in a meta-analysis of glyphosate exposure (RR 1.45, 95% CI: 1.1–1.9) (76). Additionally, results from the Agricultural Health Study showed that licensed pesticide applicators had a higher incidence of prostate cancer (SIR 1.14, 95% CI: 1.05–1.24) (77).

##### 2.5.1.3 Household air pollution in women engaged in home-based livelihoods

Lung cancer is closely linked to burning coal and biomass fuels for heating and cooking, especially in women. Pooled odds ratios for coal and biomass are 1.82 (95% CI: 1.60–2.06) and 1.50 (95% CI: 1.17–1.94), respectively, according to meta-analyses (78, 79). Women who used smoky coal in Xuanwei, China, had an absolute lifetime lung cancer mortality risk of 18–20%, and their hazard ratios were nearly 99 times higher than those of women who used smokeless coal (80, 81).

##### 2.5.1.4 Artisanal and small-scale gold mining

A significant neurotoxic and systemic danger, mercury is employed extensively in ASGM. IARC classifications, however, indicate that elemental and inorganic mercury are not classifiable (Group 3) and that methylmercury compounds are potentially carcinogenic (Group 2B) (82, 83). As a result, in ASGM settings, cancer links are secondary to more extensive toxicological effects and are hence indirect.

Policy interventions must combine legislation, engineering controls, worker empowerment, and health system integration to address the wide range of occupational hazards in traditional livelihoods. A balanced approach is necessary to ensure uptake in settings with limited resources. While bans and limitations decrease the most hazardous exposures, they must be accompanied by workable alternatives, PPE finance, and culturally appropriate training. Participatory monitoring, medical surveillance, and worker cooperatives can all help to improve accountability and enforcement. A variety of alternatives that LMICs can modify based on local requirements, capabilities, and cultural contexts are shown in **Table 5** below.

**Table 5:** Policy menu for high-risk traditional occupations.

Policy lever	Examples for LMIC traditional sectors	Additional information
Engineering controls	Wet-cutting to suppress silica dust; improved stoves and chimneys; ventilated pesticide mixing areas	Stove-improvement trials in Xuanwei reduced lung cancer risk (81)
PPE financing & access	Subsidised respirators, gloves, goggles; community PPE banks	Uptake is higher when linked to cooperatives
Worker organisation	Support cooperatives in mining/farming; participatory safety monitoring	Improves enforcement and bargaining power
Medical surveillance	Screening for silicosis/TB; registry-based pesticide exposure tracking	Simplified paper-to-digital monitoring for rural workers
Regulatory bans/restrictions	Restrict hazardous pesticides (e.g., organophosphates, paraquat); phase out smoky coal; implement Minamata-aligned mercury reduction in ASGM	Requires affordable substitutes to avoid unsafe, informal alternatives

ASGM, Artisanal and Small-Scale Gold Mining; LMIC, LMICs, Low- and middle-income countries.

#### 2.5.2 Cultural practices and household air pollution

In LMICs, household air pollution (HAP) from traditional cultural behaviors is a substantial but often under-recognized contributor to cancer risk. Wood, charcoal, crop leftovers, and dung are examples of biomass fuels that are still widely used for cooking and heating, particularly in low-income urban and rural homes, where modern fuels are either unavailable or prohibitively expensive (72, 84). Cooking techniques and fuel selection are heavily influenced by cultural norms. Open-fire stoves and other traditional cooking appliances are practical and have social and cultural value in many LMICs. For instance, some cuisines require lengthy simmering or smoking methods to produce the desired flavors. Subsequently, this encourages the use of biomass stoves despite the health dangers (85). Furthermore, women are typically in charge of cooking and gathering fuel in many cultures, which puts them and their children at a higher risk of long-term exposure to indoor pollutants (86). Many of the pollutants produced by burning biomass are known to induce cancer in humans, including carbon monoxide, volatile organic compounds, fine particulate matter (PM2.5), and polycyclic aromatic hydrocarbons (PAHs) (70, 87). According to epidemiological studies, HAP is strongly linked to an elevated risk of lung cancer, especially in women who spend a lot of time near traditional stoves (85). Furthermore, PAHs produced by biomass smoke have been associated with upper aerodigestive tract malignancies. This has led to a high regional burden of esophageal cancer in some regions of Asia and Africa (88).

In many LMICs, the adoption of cleaner cooking methods remains sluggish despite these known hazards. Aside from financial limitations, other obstacles include deeply ingrained cultural preferences and skepticism regarding contemporary cooking stoves. The obstacles are thought to be incompatible with customary cooking methods (84, 89). According to Rehfuess et al. (89), interventions that only address technological solutions without considering cultural factors have frequently failed to achieve sustained acceptance (89). Furthermore, household air pollution interacts with other socio-environmental factors rather than happening alone. In addition to low literacy and a lack of health information, poor ventilation in traditional households increases exposure to the carcinogenic risks associated with biomass smoke (72, 85). Transitions to safer energy options are frequently hampered by women’s limited decision-making authority within families, which perpetuates exposure and illness risk cycles (86). Integrated approaches that consider gender dynamics, economic realities, and cultural settings are needed to address home air pollution as a cancer risk factor in LMICs. Subsidized access to clean fuels, culturally relevant stove designs, community education initiatives, and gender-sensitive interventions that enable women to have a say in family energy choices should be included in policy measures. The burden of HAP-related cancers in LMICs can only be successfully decreased by using comprehensive and culturally sensitive approaches.

Interventions that substitute genuinely clean fuels like electricity, ethanol, or LPG for conventional open-fire cooking usually result in significant decreases in PM_2_ exposures in households, as long as the supply is consistent and use is exclusive. In Rwanda, for example, homes with an upgraded Save 80 cookstove saw a 77% decrease in indoor PM_2.5_, as well as 50% and 78% decreases in black and brown carbon, respectively (90). Tailored enhanced biomass stove implementations decreased PM_2.5_ exposures by 31%, 32%, and 65% in Uganda, Vietnam, and Kyrgyzstan; however, levels remained above WHO standards (91). According to Dillon et al. (92), “improved” biomass stoves frequently provide moderate reductions in PM_2.5_ (e.g., ~40 to 50%), which may still surpass acceptable standards (92). There is currently no direct proof that cookstove interventions lower the risk of cancer since there are no long-term randomized trials with cancer endpoints. Nonetheless, PAHs formed from biomass smoke and PM_2.5_ are recognized carcinogens that are causally associated with lung cancer (93, 94). Therefore, large, sustained reductions in PM_2.5_ could result in lower long-term risk for lung and upper aerodigestive cancers, according to mechanistic plausibility and exposure-response models (94). However, “stacking”—the practice of using conventional stoves in addition to cleaner ones—occurs often and compromises the effectiveness of exposure reductions (95). The biggest obstacles to exclusive usage include cultural preferences, stove maintenance, fuel costs, sporadic supply, and affordability (91). It is essential to design culturally suitable stoves, undertake behavior-change programs, ensure supply infrastructure, and offer subsidies to achieve the sustained, exclusive use required for significant cancer-risk reduction. Transitions to genuinely clean fuels (such as LPG and electricity) should be prioritized from the perspective of cancer prevention policy. These transitions should be backed by supply and financing strategies and implemented in a culturally appropriate manner. Only then will the significant and long-lasting reductions in PM_2.5_ that are necessary to realistically lower long-term cancer risk be achievable. Furthermore, to measure direct cancer outcomes, long-term cohort surveillance connected to intervention programs is required.

#### 2.5.3 Environmental pollution and traditional lifestyles

The threat of environmental pollution to public health has grown in LMICs, where conventional lifestyles frequently collide with new ecological hazards, increasing the risk of cancer. Although many traditional practices were historically sustainable, ecological balances have been disrupted by globalization, industrial encroachment, and lax regulatory systems. Consequently, communities are now exposed to dangerous pollutants through soil, water, air, and food systems (96, 97). Owing to land tenure insecurity and economic marginalization, traditional communities are often located next to landfills, industrial sites, urban growth zones, or contaminated waterways in LMICs. Groups that depend on subsistence, the IARC has designated heavy metals (such as arsenic, lead, and mercury), persistent organic pollutants, and agrochemical residues as carcinogenic. Agriculture and fishing may unintentionally consume water and food contaminated with these substances (98, 99). For instance, skin, lung, and bladder cancers have been connected to long-term exposure to groundwater containing arsenic, a phenomenon that has been extensively documented in South Asia (98, 100).

Conventional food storage and preservation methods also increase exposure to environmental carcinogens. Poor post-harvest management of grains and nuts promotes fungal development in many African and Asian contexts, contaminating food with aflatoxins, which are strong hepatocarcinogens associated with liver cancer (23). Chronic exposure is increased in situations when food instability deters people from throwing out tainted food, exacerbating these dangers. Furthermore, new toxic exposure vectors have been brought about by the growth of low-wage manufacturing, artisanal mining, and informal recycling. All these are frequently seen as adaptations of traditional livelihoods. Dioxins and PAHs, for example, are released into residential surroundings by informal e-waste processing, impacting populations with limited means of reducing exposure (101). Similarly, the use of lead-based glazes or fuels in traditional metalwork or ceramics leads to soil and air pollution.

Many traditional societies are unaware of the long-term health effects of environmental pollution, especially its connection to cancer, despite these exposures. The systemic underestimation of environmental carcinogens as public health concerns in LMICs is a result of both insufficient public health messaging and limited environmental monitoring (96, 102). Furthermore, traditional lifestyles continue to be extremely vulnerable to environmental degradation because of a combination of overlapping poverty, poor infrastructure, and lax enforcement of policies. Integrated, community-engaged strategies are needed to reduce the cancer risk associated with environmental pollution in conventional settings. Environmental health education, culturally relevant risk communication, encouragement of safe farming and food storage methods, and legislative frameworks that prioritize pollution control in underserved rural and periurban areas are a few examples. Sustainable health improvements in culturally embedded LMIC communities can only be attained by coordinating environmental justice and cancer prevention.

In LMICs, where exposure to environmental pollution frequently happens through a variety of channels, including soil, water, air, and food, communities that continue traditional lifestyles are at serious risk for developing cancer. Key contaminants, their main routes of exposure, the cancer outcomes linked to them, and typical regions where these hazards have been reported are compiled in **Table 6**. Along with highlighting both well-known risks, like aflatoxins and arsenic, and new or under-monitored contaminants, like POPs, PAHs, and heavy metals from artisanal practices, the table also shows the scope of national monitoring for food-chain exposures and offers a qualitative evaluation of data quality.

**Table 6:** Environmental pollution hotspots in traditional LMIC lifestyles.

Pollutant/hazard	Exposure pathway	Implicated cancer(s)	Example regions/practices	National monitoring of food-chain exposures?	Data quality/notes	References
Aflatoxins	Contaminated grains/nuts	Liver	Sub-Saharan Africa, Southeast Asia	Usually monitored in staple crops; national data exists but uneven coverage	High for liver cancer linkage; exposure assessment variable	(23)
Mercury (Hg)	Fish consumption; small-scale gold mining	Kidney, liver, possibly oral cavity	Artisanal gold mining in Ghana, Peru	National fish surveys inconsistent; subsistence fish often unmonitored	Moderate; biomonitoring sparse	(103)
Lead (Pb)	Soil, air, dust; traditional metalwork, ceramics	Kidney, neurological impacts (possible link to cancers under investigation)	Informal mining zones, artisanal pottery villages in Africa & South Asia	Rarely monitored in food-chain; mostly occupational/environmental	Moderate; exposure measured, cancer links less quantified	(104)
Arsenic (As)	Groundwater ingestion	Skin, lung, bladder	Bangladesh, India, parts of Nepal	Partially monitored (some national water surveys; food-chain data limited)	High for water contamination; limited for dietary exposures	(105)
Agrochemicals (pesticides, herbicides)	Food, water, occupational contact	Non-Hodgkin lymphoma, prostate, possibly others	Subsistence farming in LMICs	Partially monitored in water; food-chain data limited	Moderate; strong occupational evidence, limited population-level dietary data	(106)
Polycyclic Aromatic Hydrocarbons (PAHs)	Air, soil, occupational exposure (e.g., informal e-waste, charcoal burning)	Lung, skin, bladder	Informal recycling, traditional cooking methods, low-wage manufacturing	Not typically monitored in food; occupational exposure occasionally tracked	Moderate; exposure levels measured, cancer association inferred	(107)
Persistent Organic Pollutants (POPs, e.g., dioxins, PCBs)	Air, soil, food (fish, animal fat)	Liver, breast, possibly hematologic	Informal e-waste recycling sites (Ghana, India, China)	Rarely systematically monitored in food; mostly environmental sampling	Low–moderate; few population-level studies	(108)

LMICs, Low- and middle-income countries.

### 2.6 Luxury chemicals and chronic carcinogen exposure in LMICs

Chronic exposure to carcinogenic or endocrine-disrupting chemicals contained in common consumer products,often referred to as “luxury chemicals”,is an increasing worry in addition to the well-known environmental and occupational risks. These include cosmetics, air fresheners, perfumes, personal care items, and home insecticides. Although these items have historically been used in high-income environments, urbanization, growing affluence, and changing consumer preferences for modern lifestyles are driving a sharp increase in their use in LMICs (109). Low public understanding of the possible health concerns presented by the chemicals used in consumer products is a major issue in LMICs. Phthalates, parabens, formaldehyde-releasing preservatives, and synthetic fragrances are chemicals found in many commercial products. These substances have been linked to endocrine disruption and an increased risk of cancer, especially hormone-dependent cancers such as breast and prostate cancer (**Table 7**) (132, 133). The biological load of potentially carcinogenic compounds can increase over time due to chronic exposure, even at low levels, particularly in women who may use many personal care products daily (134).

**Table 7:** Risk factors, associated cancer sites, and strength of evidence.

Risk factor/exposure	Associated cancer sites	Key references
Dietary risks		
- Aflatoxins (contaminated maize, groundnuts)	Liver (Hepatocellular carcinoma)	(110, 111)
- Salted/preserved foods	Stomach	(112)
- Red and processed meat	Colorectal	(113, 114)
- Low fruit and vegetable intake	Esophageal, gastric, colorectal	(115, 116)
Traditional and complementary medicine (T&CM)		
- Herbal preparations (unregulated)	Liver, kidney, hematologic (toxicity, contamination)	(117, 118)
- Delayed diagnosis due to T&CM use	Multiple sites (indirect effect)	(42, 119)
Tobacco		
- Smoking (cigarettes, bidis, hookah)	Lung, oral cavity, larynx, bladder, pancreas	(120, 121)
- Smokeless tobacco (betel quid, gutkha, snus)	Oral cavity, pharynx, esophagus, pancreas	(122, 123)
Alcohol		
	Oral cavity, pharynx, larynx, esophagus, liver, colorectum, breast	(124, 125)
Occupational exposures		
- Asbestos	Lung (mesothelioma)	(126)
- Pesticides	Leukemia, lymphoma, prostate	(127)
- Mining (silica, uranium, radon)	Lung, stomach	(128)
- Biomass fuel smoke	Lung	(85, 129)
Luxury chemicals/consumer products		
- Cosmetics, skin-lightening creams (arsenic, mercury, hydroquinone)	Skin, kidney	(63, 130)
- Endocrine-disrupting chemicals (plastics, cosmetics)	Breast, prostate, testicular	(127, 131)

Another important form of exposure is the use of household insecticides. Due to cramped living circumstances and worries about vector-borne diseases, pesticides are commonly used in LMICs for both residential and agricultural pest management. Many of these products are readily accessible with little regulatory monitoring or user education regarding safe handling and ventilation, even though they include organophosphates, pyrethroids, and other substances associated with hormone disturbance and cancer risk (135). Similarly, air fresheners and air conditioners can emit known or suspected carcinogens called volatile organic compounds (VOCs), including formaldehyde, toluene, and benzene (136). The dangers of inhalation exposure are increased by inadequate ventilation in both traditional and urban houses, especially in hotter areas where the use of air conditioning is growing rapidly. Over decades, cumulative exposure to chemicals from many sources, such as cleaning products, cosmetics, fragrances, and household fumigants, may be minor but substantial, perhaps causing hormonal imbalances and cancer development (132). However, regulatory frameworks are frequently lacking, disjointed, or insufficiently enforced, and knowledge of these hazards is low in LMICs. Many LMICs lack comprehensive rules regulating the disclosure of ingredients, safety testing, or labeling of hazardous chemicals in consumer goods (96, 99).

Limited technical capability for chemical monitoring, conflicting health goals, and financial demands from expanding consumer product markets are obstacles that policy solutions in LMICs must overcome. However, a few crucial steps must be taken.

- Develop regulatory standards aligned with global frameworks (e.g.,SAICM) for the safe limits of carcinogens and endocrine disruptors in consumer goods (99).
- Launch culturally tailored public education campaigns that emphasize safer alternatives and promote practical strategies to minimize exposure (133).
- Support product reformulation and transparent labeling by incentivizing manufacturers to reduce hazardous ingredients and disclose chemical content (134).
- Increased investment in epidemiological research is required to assess exposure levels, health outcomes, and locally relevant mitigation strategies.

Without prompt action, the growing use of luxury chemicals in LMICs poses the potential to create new cancer risk pathways, compounding the cost of occupational and environmental exposures and making cancer preventive methods even more challenging.

In LMICs, the increasing use of consumer goods that contain endocrine-disrupting or carcinogenic chemicals indicates a shift in cancer risk from historic exposures to contemporary lifestyle risks. In contrast to food or occupational exposure, these dangers are frequently cumulative, imperceptible, and poorly understood by regulators and communities. According to the analysis, this emphasizes the necessity of public awareness campaigns, regulatory capacity building, and improved global-local coordination to stop luxury chemicals from emerging as the next major cause of avoidable cancer risks in LMICs.

Together, these cultural customs, the use of traditional medicine, and environmental exposures show how sociocultural norms, structural injustices, and livelihood realities interact to create a cancer risk in LMICs. In contrast to high-income settings, where cancer prevention frequently emphasizes lifestyle modification alone, LMICs face overlapping and context-specific drivers, such as unsafe food storage, reliance on unregulated T&CM, widespread alcohol and tobacco use, hazardous occupations, and increased exposure to modern consumer chemicals. These hazards are cumulative rather than isolated, making vulnerable groups, especially women and laborers in the unorganized sector, even more vulnerable. Analytically, this emphasizes that more than biomedical solutions are required for effective cancer prevention in LMICs. Instead, integrated strategies that address cultural legitimacy, control harmful exposures, and integrate cancer control into the larger agendas of social justice, labor protection, and environmental health are needed.

**Table 8** below outlines significant consumer product categories that contribute to long-term exposure to potentially harmful chemicals in LMICs. It classifies cosmetics, fragrances, household insecticides, and cleaning agents, identifies the primary chemical classes they contain, including endocrine-disrupting chemicals (EDCs), volatile organic compounds (VOCs), and metals. Furthermore, it emphasizes the associated health risks, particularly hormone-dependent and other cancers. Verified references are provided to support the reported relationships and demonstrate the cumulative nature of exposure to daily luxury compounds.

**Table 8:** A summary of product categories, common chemical classes, and associated health hazards.

Product category	Common chemical classes	Examples of chemicals	Potential health impacts/cancer risks
Fragrances	EDCs, VOCs	Synthetic fragrances, phthalates, formaldehyde, toluene, benzene	Endocrine disruption, cumulative cancer risk via inhalation
Cleaning Agents	VOCs, Preservatives	Formaldehyde, benzene, toluene, solvents	Hormonal imbalance, long-term cancer risk via cumulative exposure
Household Insecticides	EDCs, Pesticides (organophosphates, pyrethroids)	Malathion, chlorpyrifos, permethrin	Hormone disturbance, leukemia, lymphoma, prostate cancer
Cosmetics & Personal Care	EDCs, Metals	Phthalates, parabens, formaldehyde-releasing preservatives, arsenic, mercury, hydroquinone	Hormonal disruption, breast, prostate, testicular cancers; skin & kidney toxicity

EDCs, endocrine-disrupting chemicals; VOCs, volatile organic compounds.

## 3 Sociocultural barriers to cancer prevention and early detection

### 3.1 Cancer myths

Persistent misconceptions about cancer impede attempts at prevention, early identification, and treatment in many LMICs. These myths, which result in poor health outcomes and delayed medical care, are frequently based on cultural beliefs, spiritual interpretations, and false information (137). It takes specialized, culturally aware therapies that connect conventional wisdom with scientifically supported medical knowledge to dispel these myths.

The idea that cancer is a spiritual or supernatural disease, delivered as a curse or punishment, and that it can only be cured by religious or traditional methods, is a prevalent misconception in many LMIC groups. Patients who have such views frequently put off receiving medical care in favor of speaking with traditional healers or religious leaders (40, 138). Integrating spiritual and traditional viewpoints into health education can help combat this. For instance, the NGO mothers2mothers works with community leaders and local moms in Malawi to encourage HPV vaccination among girls between the ages of nine and 18. Their method dispels cultural myths while presenting accurate data, proving that cancer preventive strategies align with regional beliefs (139).

The idea that cancer always results in death is another common fallacy. People are discouraged from obtaining timely care because of this notion, which breeds fear, social isolation, and stigma (138). Highlighting survivor experiences and stressing the value of early detection and treatment are examples of counterstrategies. To spread correct information about the HPV vaccine and cervical cancer, community-based campaigns in Kisumu County, Kenya, involve local leaders and healthcare professionals. These programs have boosted the uptake of preventative measures and effectively decreased stigma (137). There are still misconceptions regarding the causes of cancer, with some populations attributing the disease to physical damage, sexual promiscuity, or the use of contraception instead of biological or environmental factors (140). Education about the true causes of cancer, such as the part oncogenic viruses play in cervical cancer, is necessary to address this. Programs in Uganda that promote HPV vaccination aim to dispel these myths by reaffirming that cancer results from observable biological causes rather than from moral or behavioral transgressions (137).

Last but not least, misconceptions about HPV vaccination, such as worries about infertility or alleged harmful side effects, present further obstacles to prevention. Building faith in vaccine safety requires the dissemination of evidence-based information via reliable community channels. Research on parental and adolescent concerns over HPV vaccination in Kisumu County has influenced community engagement tactics that promote trust and dispel myths, leading to increased vaccination uptake (137). Together, these instances highlight the value of community-driven, culturally sensitive strategies in debunking cancer stereotypes. By involving reliable people like survivors, religious leaders, and traditional healers, health initiatives can successfully reframe cancer as a disease that can be prevented and treated, encouraging prompt medical attention and enhancing results in LMICs.

### 3.2 Cancer stigma

**Figure 5:**
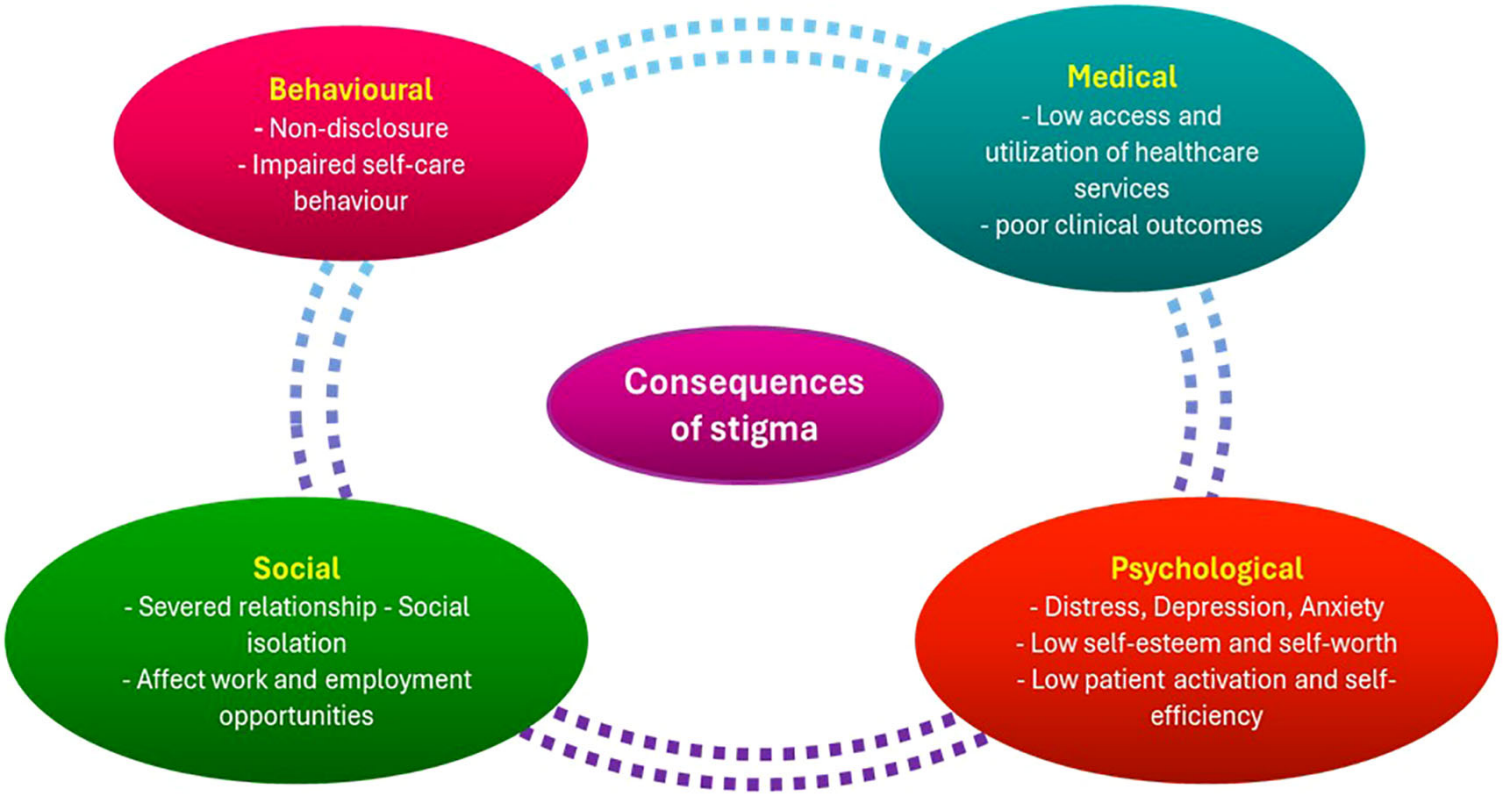
Consequences of stigma in patients with cancer. The diagram illustrates how stigma exacerbates worse survival outcomes, social isolation, treatment noncompliance, and delays in seeking care. It highlights how crucial it is to incorporate stigma reduction techniques into initiatives aimed at preventing and controlling cancer. This is an original figure.

Effective cancer control is severely hampered by cancer stigma in LMICs, where patient experiences and public perceptions are shaped by a confluence of institutional injustices, cultural beliefs, and false information. Rather than being a personal issue, stigma has a broad impact on health-related behaviors, healthcare systems, and community dynamics, which eventually leads to preventable morbidity and mortality (40, 141). At each stage of the patient journey, stigma shapes health-seeking behavior and acts as a powerful barrier to cancer care. Shame and prejudice can make it difficult for people to stick with therapy, and fear of disclosure and social rejection frequently keeps people from coming in early. These effects are summarized in **Figure 5**, which illustrates how stigma results in delayed diagnosis, treatment discontinuation, dependence on non-biomedical therapy, and ultimately worse survival outcomes. This demonstrates that stigma is a structural factor that contributes to disparities in cancer outcomes among LMICs in addition to being a psychosocial problem. In many LMICs, cancer is viewed as a moral failure or supernatural retribution, which encourages social marginalization and secrecy (138). For instance, cancer is frequently linked to curses or “evil spirits” among the Acholi population in northern Uganda, leading patients to turn to traditional healers rather than medical facilities for treatment (40).

Anticipated stigma and fear of others’ reactions deter individuals from revealing symptoms, seeking screening, or following treatment plans (138). Since breast and cervical cancer are linked to ideas of sexual appropriateness, femininity, and reproductive worth, the stigma surrounding these diseases can be especially strong among women with them. Due to this stigma, women frequently hide their symptoms or postpone potentially life-saving medical care out of fear of social rejection or abandonment. These difficulties are exacerbated by institutional problems. Insufficient psychosocial support services, inappropriate communication from medical staff, and lack of privacy in healthcare settings all lead to patient disengagement and stigma reinforcement (22). These institutional flaws can exacerbate mistrust and alienation in LMIC, where healthcare services are typically overworked and underfunded. Stigma hinders adherence to therapy, causes delays in diagnosis, and lowers participation in cancer screening. Additionally, as patients retreat from their jobs or social lives to hide their disease, it exacerbates psychological suffering, lowers their quality of life, and may result in financial difficulty (141). Comprehensive and culturally aware interventions are necessary to combat cancer stigma in LMICs. Effective tactics include using cancer survivors as champions, incorporating stigma-reduction messaging in public health initiatives, and enlisting the help of religious and community leaders to dispel myths (142). Furthermore, to prevent clinical interactions from unintentionally reinforcing stigma, healthcare professionals must be trained in compassionate, patient-centered communication (22). Importantly, interventions must be created to empower underrepresented groups and be customized to local cultural contexts.

#### 3.2.1 Diagnostic delays associated with cancer stigma

In LMICs, the stigma associated with cancer harms patient outcomes by greatly contributing to delays in diagnosis. According to a study conducted in Libya, the median interval between the onset of symptoms and the diagnosis of breast cancer was 7.5 months, with 56% of patients receiving a diagnosis more than six months later. According to Mwaka et al. (40), these delays were induced by several factors, including the belief that symptoms were not significant (27%), dependence on alternative therapies (13%), and fear or embarrassment that prevented medical consultation (10% and 4.5%, respectively) (143). A mixed-methods study conducted in India found that the median main delay for diagnosing breast cancer was 86 days, whereas the median secondary delay was 23 days. According to the study, a family history of smoking or tobacco use was linked to secondary delay, whereas characteristics including housing style and the value placed on health were linked to primary delay (144). According to an Iranian study, 16.4% of women with breast cancer postponed getting treatment for more than 90 days, while 34.3% did so for 30 to 90 days. A 5.7-fold increased risk of delaying seeking help was linked to the stigma around breast cancer (145).

#### 3.2.2 Interventions to reduce diagnostic delays

Multifaceted interventions that are adapted to the cultural and social circumstances of LMICs are necessary to address cancer stigma and the delays that are linked to it. Campaigns for community-based awareness have been successful in lowering stigma and encouraging early diagnosis. For example, 16 out of 19 general population-focused treatments that focused on community participation, lowering the stigma associated with cancer, and raising general health awareness were found in a systematic review (146). Additionally, educating medical professionals in patient-centered, compassionate communication can help reduce stigma in clinical settings and create a welcoming atmosphere that promotes prompt medical consultation. In healthcare institutions with limited resources, technological developments like the creation of resource-efficient diagnostic tools, like MobileNetV2-based models, present intriguing options for early cancer detection. The difficulties caused by protracted diagnosis delays in underdeveloped nations have been addressed by these models, which have shown excellent diagnostic accuracy and efficiency (146).

In summary, the stigma associated with cancer serves as an example of how institutional flaws and societal attitudes combine to perpetuate disparities in treatment and prevention of the disease. In addition to silencing people, stigma damages public confidence in healthcare systems, which exacerbates delayed diagnoses and inadequate treatment compliance. To achieve equitable cancer control in LMICs, our analytical conceptualization demonstrates that stigma reduction must be viewed as a systemic intervention that calls for community-based lobbying, survivor visibility, and professional health training for compassionate communication.

### 3.3 Case studies: the influence of socio-cultural norms on cancer epidemiology

Sociocultural norms significantly impact cancer epidemiology in LMICs. They influence how people interact with health institutions as well as how disease occurrence patterns are shaped. Case studies from various LMIC contexts show how differences in cancer risk, diagnosis, and outcomes are influenced by a combination of cultural beliefs, gender dynamics, economic realities, and traditional practices in these countries. The high incidence of cervical cancer in SSA is a prime example of how cultural norms and cancer epidemiology interact with each other. Although cervical cancer can be prevented through screening and immunization, honest communication about gynecological symptoms is impeded by cultural taboos surrounding modesty, sexuality, and reproductive health. According to studies conducted in Nigeria and Uganda, many women avoid cervical cancer screening because they are ashamed of pelvic exams, fear stigma, or believe that cervical cancer is a sign of promiscuity or divine retribution (40, 138). Cervical cancer has a high mortality rate because it is frequently detected at an advanced stage.

Similar dynamics are observed in South Asia, where cultural perceptions of breast cancer significantly impact diagnosis and care. Women in countries such as India and Pakistan often delay seeking medical help for breast lumps or symptoms. This is because breast cancer is seen as a personal or embarrassing matter. These delays are exacerbated by economic reliance on male family members, marital instability and fear of social rejection (140). Consequently, a significant proportion of South Asian women present with advanced breast cancer despite increased awareness efforts. Southeast Asian cancer risk patterns are influenced by regionally specific dietary and customary behavior. Chewing betel quid is a common habit closely associated with social customs, hospitality, and prestige, especially in Myanmar, Cambodia, and some regions of Vietnam. However, research indicates that habitual users have a two- to seven-fold higher risk of developing oral and pharyngeal malignancies as a result of this activity (57, 67). Cultural ties to betel quid are still strong despite public health messaging, which makes efforts to avoid cancer more difficult.

Another example of how sociocultural factors impact cancer epidemiology is in Latin America. Traditional indoor wood-burning stove-based cooking techniques in some indigenous cultures raise household air pollution levels, which are known risk factors for upper gastrointestinal and lung malignancies (85, 88). Cultural preferences for conventional cooking methods and reluctance to adopt new technologies have frequently caused attempts to implement cleaner cooking technologies to fail (89). Furthermore, gender norms are significant factors in the epidemiology of cancer in many LMICs. Due to the belief that talking about health issues diminishes masculinity, men may postpone treatment for diseases such as prostate or colorectal cancer. Gendered reluctance has resulted in poorer survival rates and advanced-stage diagnoses. Together, these case studies demonstrate that biological variables alone are insufficient to explain cancer epidemiology in LMICs. Cultural norms, social roles, economic pressures, and customs significantly impact who is in danger, who seeks treatment, and who survives cancer. Gender-sensitive, culturally appropriate, and community-involvement-based interventions are necessary to address these inequities. Cancer management initiatives in LMICs run the risk of being ineffective or even detrimental if sociocultural contexts are not recognized and integrated (22, 142).

When considered as a whole, these case studies show that biological risk factors alone are insufficient to explain cancer epidemiology in LMICs. Economic limitations, gender norms, and cultural traditions continuously influence vulnerability, care, and survival. In terms of analysis, this emphasizes the necessity of medically effective therapies that are also gender-sensitive, culturally appropriate, and based on community involvement to lessen cancer disparities within LMICs.

Overall, the prevalence of stigma, myths, and culturally specific health-seeking practices related to cancer in LMICs shows that biological advancements alone cannot enhance outcomes without concurrent sociocultural engagement. Late diagnosis and low survival rates are directly caused by stigma that suppresses symptoms and inhibits disclosure, as well as misconceptions that portray cancer as supernatural or incurable. Case studies from Asia, Latin America, and Africa also show how economic reliance, cultural taboos, and gender norms reinforce these barriers, influencing not only who gets cancer but also who receives timely care. Analytically, these findings highlight the need for community-led advocacy, stigma reduction, and culturally grounded communication as key preventive measures for cancer control in LMICs. Health systems can create the trust and cultural resonance required to change cancer from a feared and concealed disease to one that is publicly discussed, prevented, and curable by redefining it within local belief systems and elevating trusted community voices.

### 3.4 Health system mistrust and culturally safe care models

One of the biggest obstacles to early cancer detection and treatment in LMICs is mistrust of the official health system. People may be discouraged from using biomedical services due to historical injustices, bad experiences in the past, and perceived cultural insensitivity (22). Fears of discrimination, expensive out-of-pocket expenses, and a lack of knowledge about medical procedures frequently exacerbate this mistrust, which leads to delayed presentation and poor treatment protocol adherence (138).

By developing health services that are sensitive to patients’ social, cultural, and language needs, culturally safe care models have been demonstrated to lessen these obstacles. Peer educators, language-concordant counseling, and patient navigation programs are among strategies that enable patients to participate in care while honoring their cultural background (142). For example, peer educators and community health professionals have been used in Kenya and Uganda to offer advice on HPV vaccination and cervical cancer screening. These programs have raised awareness, boosted the use of preventative treatment, and bolstered confidence in official medical professionals (40, 137).

Overall, including culturally acceptable practices into cancer prevention and care can reduce delays, improve adherence, and establish a long-term link between communities and health institutions. These approaches are crucial for obtaining equitable cancer outcomes in LMICs.

## 4 Cancer prevention strategies in LMICs

**Figure 6:**
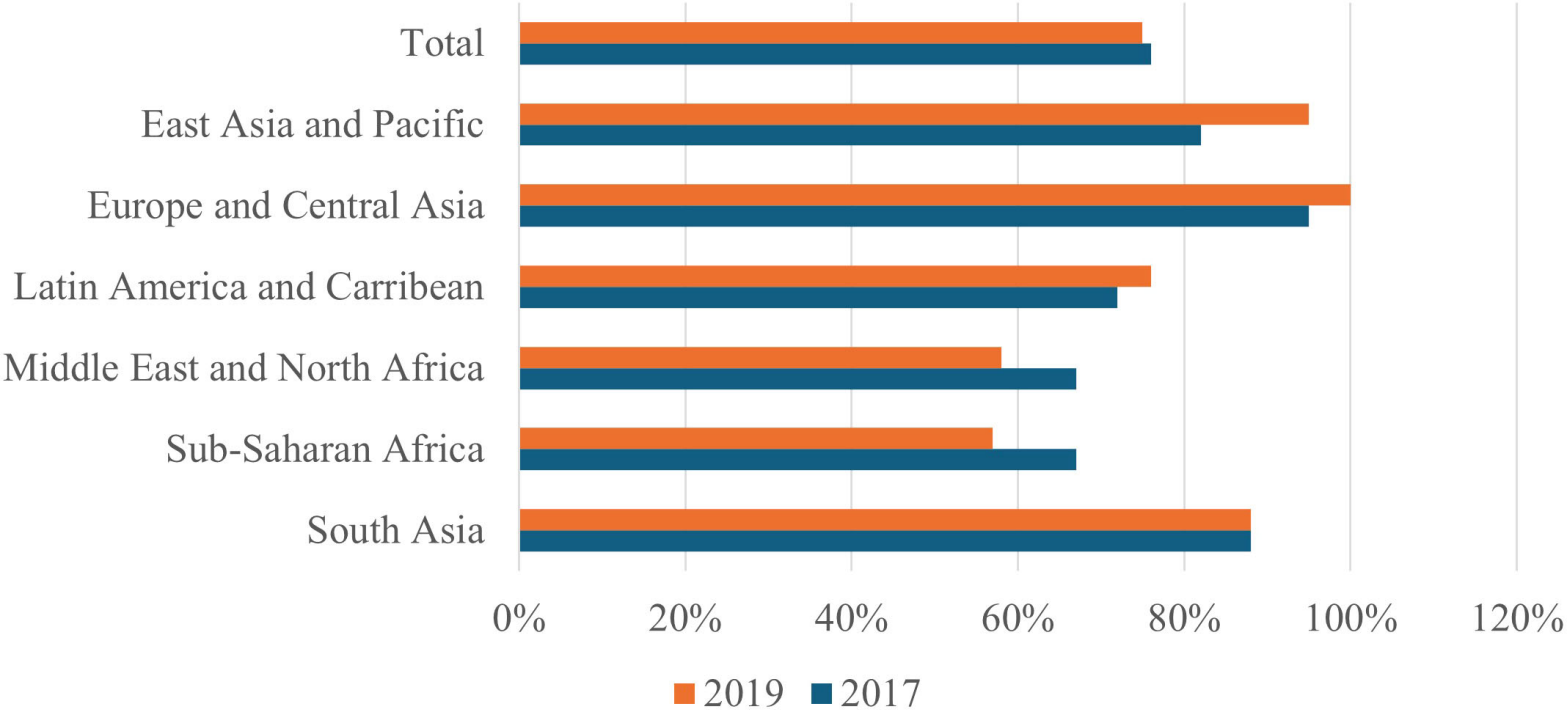
Percentage of LMICs that had core cancer control components such as cancer policies and plans in 2017 and 2019. The figure illustrates the disparities in policy implementation, with many LMICs lacking palliative care, screening programs, and registries. It highlights the need for more system-level investment and integrated policies to combat the rising cancer burden. LMICs, Low- and middle-income countries. This is an original figure.

It is evident from the sociocultural constraints discussed in Section 3 that biological approaches alone are insufficient for cancer prevention in LMICs to be effective. Early detection and treatment are frequently undermined by myths, stigma, and culturally rooted practices that influence the perception of risk factors and the pursuit of care. Therefore, preventive strategies must be both culturally sensitive and scientifically sound. This section describes the multilevel strategies that directly address the structural, cultural, and environmental factors influencing cancer risk in LMICs. These strategies cover policies, education, technology, and health system integration. A plan to address cancer’s place in the global health agenda with other NCDs was created in 2011 during a high-level conference of the United Nations (UN) General Assembly on NCD Prevention and Control (147). The report warned that many LMICs will not have adequate funding for comprehensive cancer management strategies, making them ill-prepared to handle the anticipated rise in the number of patients with cancer. Their report emphasized the growing concerns regarding the increase in the cancer burden in LMICs (148, 149). Since the UN General Assembly meeting, some progress has been achieved. For example, in 2017, 101 of the 133 LMICs had a strategic action plan or operational policy for the treatment and prevention of cancer. However, few have made specific budgetary commitments to carry out such plans (3, 150). This figure plateaued at 100 out of 133 LMICs by 2019, with declines in SSA and the Middle East and North Africa (**Figure 6**). **Figure 6** demonstrates stagnation in policy adoption, reinforcing that policy implementation is not only a technical issue but also a political and cultural challenge that requires multilevel advocacy.

Multilevel, culturally sensitive strategies that consider the distinct risk landscapes and social contexts of LMICs are necessary for cancer prevention. A complex interaction of behavioral, environmental, and sociocultural factors is driving the rise in cancer incidence in LMICs, necessitating solutions beyond merely biological interventions (54, 70). Misinformation and stigma often impede grassroots prevention efforts. People are discouraged from undergoing screening or reporting symptoms. This is due to deeply ingrained cultural ideas that frequently depict cancer as a contagious disease, punishment, or death sentence (138, 151). Research has demonstrated that community-based education programs can successfully dispel false beliefs and increase the rate of early diagnosis. This is particularly true when they are presented through reliable sources such as religious leaders, traditional healers, and cancer survivors (22, 142). Behavioral risk reduction is a key component of preventative initiatives. Reducing the risk of cancer requires campaigns that target alcohol and tobacco use, encourage physical activity, and promote healthier diets (54, 152). However, in LMICs, where poverty, urbanization, and cultural norms frequently sustain high levels of risk behaviors, such as the use of smokeless tobacco and the consumption of processed foods, these treatments encounter challenges (153). Environmental and occupational dangers introduce an additional degree of complication. Carcinogens, such as biomass smoke, herbicides, and industrial pollutants, are present in many traditional livelihoods (87, 96).

Regulations, safer work practices, and locally relevant education are necessary to address these dangers. However, enforcement is frequently hindered by a lack of resources and conflicting public health objectives (99). Vaccination and screening for secondary prevention are essential but are underutilized in LMICs. For instance, despite the demonstrated effectiveness of the HPV vaccine and cervical cancer screening, acceptance remains low due to financial constraints, cultural reluctance, and logistical obstacles (138, 151). Although they have potential, innovations such as HPV self-sampling and the incorporation of screening services into maternal health care need to be implemented with cultural sensitivity and include the community (99, 142). Furthermore, there are potential problems in incorporating T&CM into cancer prevention. Despite its widespread use, T&CM may cause negative interactions with biological medicines or delay diagnosis if used without proper regulation (34, 42). If supported by legislation and evidence-based recommendations, using traditional healers as collaborators in early detection and health education could close the gaps between traditional practices and contemporary care (48). The most economical way to lower the incidence of cancer and health inequalities in LMICs, where resources are frequently scarce, is prevention. To make progress, biomedical research must be combined with cultural awareness and community empowerment to create solutions that have local resonance and quantifiable effects.

### 4.1 Policies for preventing cancer in LMICs

The creation and strict application of cogent policies that address both personal risk behaviors and more general environmental and societal factors of cancer are critical for effective cancer prevention in LMICs. However, many LMICs still lack comprehensive cancer control strategies or encounter major obstacles in implementing policy commitments, even in the face of growing worldwide acknowledgment of cancer as a public health priority (54, 70). The WHO FCTC, which establishes legally binding guidelines for lowering tobacco use, a major cause of cancer globally, is a vital policy tool in the fight against cancer. Despite the FCTC being adopted by almost all LMICs, its implementation is still unequal because of industry meddling, lax enforcement, and conflicting economic priorities (55). Research indicates that price-based interventions, such as taxes, are some of the most successful strategies; for example, a 10% rise in tobacco prices results in a 4–8% decrease in tobacco use, especially among young people and those with low incomes (60). However, because of concerns about possible effects on local economies or income loss from illegal trade, many LMIC governments are still reluctant to levy large taxes (54). Another important but neglected policy tool in LMICs is the control of alcohol consumption. Although alcohol consumption is a known risk factor for several cancers, legislative measures such as taxes, sales limits, and advertising bans are not always consistently implemented. Marketing tactics aimed at women and young people make regulation even more difficult, raising the risk of alcohol-related cancer in LMIC populations (152, 154).

Occupational and environmental policies are equally important but are frequently overlooked. In LMICs, exposure to carcinogens, such as pesticides, aflatoxins, heavy metals, and biomass smoke, is a common part of many traditional livelihoods (87, 96). Although international frameworks have regulatory criteria for hazardous substances, resource limitations, lack of technical capability, and lack of political priority usually hinder national-level enforcement in LMICs (99). Many traditional economies are informal, which makes regulatory control even more difficult and leaves a sizable portion of the workforce vulnerable to exploitation. Vaccination laws are another important preventive measure. For instance, the hepatitis B vaccine is essential for lowering the incidence of hepatocellular carcinoma, and HPV immunization can prevent cervical cancer. Notwithstanding the evident cost-effectiveness and efficacy of HPV vaccination programs in reducing cancer incidence, their implementation in many LMICs has been hindered by logistical and financial obstacles (138). National cancer control plans (NCCPs) offer a vital policy framework for combining palliative care, treatment, early detection, and prevention. However, as of 2020, only 69% of nations worldwide reported having an operating NCCP, and there are notable gaps in LMIC funding and implementation (115). Plans frequently exist on paper but lack sustainable funding, meaningful roadmaps, and systems for tracking advancement (70). Ultimately, local reality and global evidence must be balanced when developing policies for LMICs. Cultural adaptability, political will, intersectoral cooperation, and sufficient funding are necessary for effective policies to go beyond verbal promises to real health benefits. Furthermore, to ensure acceptance and sustainability, policy procedures must involve communities, civil society organizations, and traditional leaders (22, 142). Cancer prevention in LMICs will continue to be dispersed and insufficient in the absence of strong, context-sensitive policies, aggravating global health inequalities and leaving millions at risk of avoidable cancers.

### 4.2 Mobile health (mHealth) utilization

Traditional healthcare delivery is frequently hampered by financial limitations and health system fragmentation in LMICs, where mobile health (mHealth) has emerged as a viable approach for cancer prevention, early diagnosis, and patient support. According to Kay et al. (155) and Piette et al. (156), mHealth is the use of mobile devices, including smartphones, tablets, and SMS services, to assist in medical and public health practices. It provides creative ways to reach underserved populations. mHealth is particularly appealing in LMICs for several reasons (155, 156). Mobile phone usage has skyrocketed in these places; even in rural areas, the GSMA reports over 90% mobile coverage (157). This technology offers a special way to distribute behavioral therapies, health information, and reminders at a reasonable cost to the user. To prevent cancer, mHealth interventions have been successful in spreading information about risk factors, encouraging screening, and dispelling enduring stigma and misconceptions about the disease (142, 158). According to data from LMICs, SMS reminders have the potential to increase screening rates. According to research conducted in Kenya and Tanzania, text messaging interventions greatly improved attendance at cervical cancer screening appointments. It has helped overcome obstacles such as logistical difficulties, low health literacy, and forgetfulness (142, 159). Similarly, mHealth techniques have been used to enhance HPV vaccination campaigns, increasing vaccine uptake and community awareness. In addition to prevention, mHealth has proven useful in helping cancer patients undergo treatment.

In situations where patients may reside far from treatment facilities, mobile applications and messaging services make it easier to monitor symptoms, schedule appointments and provide psychosocial support (156). These resources help increase adherence to treatment plans and lessen loneliness. However, several obstacles prevent mHealth from being widely used in LMIC cancer care. There are still large gender and socioeconomic gaps in mobile phone ownership and use, and digital literacy is still unequal (157). Women are frequently less likely than men to own a mobile phone or have autonomous control over its use, despite carrying a disproportionate burden of malignancies such as breast and cervical cancer (142, 157). Privacy and data security are major problems, particularly when handling sensitive health information, such as cancer diagnoses, which can carry a great deal of shame in many societies (22). Furthermore, although mHealth interventions are frequently successful in pilot projects, it is still difficult scaling up these activities sustainably remains challenging. Many programs are executed in isolation without cross-sector coordination, are dependent on temporary donor money, or are not integrated into national health systems (159). Instead of becoming transformative, mHealth runs the risk of becoming dispersed and redundant if it is not aligned with larger health policies and infrastructure. Careful planning is necessary to guarantee cultural relevance, gender equity, and integration into current health systems to implement effective mHealth initiatives for cancer prevention and care in LMICs. Digital disparities must be addressed, data privacy must be clearly defined, and sustainable financing sources must be supported by the policy frameworks. When used carefully, mHealth has great potential to close gaps in cancer care and prevention in LMICs, improving the efficiency and equity of healthcare delivery.

## 5 Conclusion

This review demonstrates how culture, environment, and social context influence cancer risk in LMICs, in addition to biological factors. Tobacco, alcohol, T&CM, traditional diets, and dangerous occupations are all factors associated with avoidable malignancies. Myths and stigma increase inequality by delaying diagnoses and treatments. Interventions must directly interact with communities outside hospitals to improve outcomes. Safer food storage and nutritional recommendations, T&CM integration and regulation, stricter enforcement of alcohol and tobacco legislation, occupational protection, and stigma reduction through reliable local voices are among the top priorities. Survivor advocacy and digital health tools can help close awareness and access gaps. Evidence from future studies must be applicable locally and actionable. Measuring environmental and occupational exposure, testing culturally appropriate remedies, and assessing T&CM safety are important gaps in the literature. Developing successful and culturally acceptable cancer prevention techniques requires a transdisciplinary approach that connects biological sciences, public health, and social sciences.
